# Identifying Unmet Social Needs in a Patient Living in Isolation: A Case Report

**DOI:** 10.7759/cureus.52429

**Published:** 2024-01-17

**Authors:** Junki Mizumoto, Yukinori Harada, Toshihiro Terui, Saori Horo, Yumi Otaka, Yuya Yokota, Masanari Komatsu, Yuko Takeda

**Affiliations:** 1 Department of Medical Education Studies, International Research Center for Medical Education, Graduate School of Medicine, The University of Tokyo, Tokyo, JPN; 2 Department of Diagnostic and Generalist Medicine, Dokkyo Medical University Hospital, Mibu, JPN; 3 Department of Neuropsychiatry, Fukushima Medical University, Fukushima, JPN; 4 Department of Nursing Care, Kinikyo Tomakomai Clinics, Tomakomai, JPN; 5 Department of Family Medicine, Family Practice Center of Okayama, Okayama, JPN; 6 Department of General Internal Medicine, Kagoshima Seikyo Hospital, Kagoshima, JPN; 7 Division of Medical Education, Faculty of Medicine, Juntendo University, Tokyo, JPN

**Keywords:** social vital signs, social determinants of health, medical communication, interprofessional collaboration, family medicine, community medicine

## Abstract

Loneliness and social isolation are common among older adults. To deliver high-quality care to older patients, healthcare professionals should know the social conditions of their patients. Addressing social determinants of health (SDH) in daily practice is beneficial to both patients and healthcare professionals. We illustrate a patient with congestive heart failure and cognitive decline whose social conditions improved through an SDH assessment. An SDH assessment has some potential advantages, which include facilitating a comprehensive understanding of patients’ social conditions, visualizing how patients’ social conditions have changed, deepening interprofessional collaboration, and ameliorating unnecessary negative emotions toward patients. This case report conveys two key messages. Firstly, healthcare professionals have the capability to evaluate patients' social backgrounds and enhance their health and social conditions through routine care. Secondly, the utilization of an SDH screening toolkit can support and enhance this initiative.

## Introduction

Loneliness and social isolation are common. Almost half of people living in Japan experience long-term loneliness, and the proportion is higher in older adults [1]. The degree of social isolation among older people in Japan is higher than in other countries and is getting worse [2]. In Japan, various factors, including the aging of the population, weakened community ties, and an increase in unmarried people, have led to a loss of social relationships among older adults [3]. Older adults in Japan have been suffering from a high prevalence of social isolation and are vulnerable to premature death [4]. Under this epidemic of loneliness and social isolation [5], Japan became the second country in the world, after the United Kingdom, to launch the Minister of Loneliness [6].

Social determinants of health (SDH), or non-medical factors that influence health outcomes (e.g., income, employment, social exclusion, housing, early life, and social gradients), have a significant impact on the health conditions of patients. In primary care settings, healthcare professionals should address the SDH of patients at regular consultations according to the data that many patients have unmet basic resource needs [7]. However, asking patients for appropriate information about their SDH, including loneliness and social isolation, remains difficult and somewhat puzzling for healthcare professionals [8].

Herein, we illustrate the case of an older man with loneliness and social isolation and discuss how healthcare professionals can address his social issues and improve his condition.

## Case presentation

An 83-year-old male with chronic heart failure with atrial fibrillation and hypertension, dyslipidemia, hyperuricemia, and insomnia received regular follow-ups at a hospital and was in stable condition. He took atenolol 12.5 mg/d, candesartan 4 mg/d, amlodipine 2.5 mg/d, furosemide 30 mg/d, spironolactone 37.5 mg/d, lixiana 5 mg/d, lansoprazole 15 mg/d, pravastatin 5 mg/d, febuxostat 10 mg/d, brotizolam 0.25 mg/d, and nitrazepam 5 mg/d regularly. He lived alone, was well dressed, and walked with a cane. He used a hospital courtesy bus to visit the hospital. However, his family physician and nursing staff noticed that, in the last year, he seemed to suffer from cognitive decline and changes in his personality, which included irritability. For example, the patient often missed appointments and frequently called the hospital to complain miscellaneously (e.g., desire to change his family physician, medication loss, general fatigue). Treatable causes of his cognitive impairment could not be detected. In addition, his edema in the lower legs got worse, and lifestyle guidance (e.g., a lower intake of sodium) did not improve it. One day, he drove to the hospital for a regular visit on his own, a task he had never undertaken before. The healthcare team was surprised as he told them that, because of his age, he had stopped driving and had begun using the hospital bus. The team considered the possibility that he needed assistance for daily living and decided to assess his social background.

First, the healthcare team tried collecting information about his social background by using a multidisciplinary approach. The team introduced the mnemonic HEALTH+P, which is widely accepted in Japan [9]. HEALTH+P stands for human network and relationships, employment and income, activities that make one’s life worth living, literacy and learning environment, taking adequate food, shelter, and clothing, healthcare system, and patient preference and personal values. Data included interviews with his son and his care manager (a care coordinator in the community appointed by Japanese long-term care insurance). The information gained from these interviews was limited by the patient’s reluctance to talk about himself when he came to the hospital. Therefore, the healthcare team visited his home to understand his living conditions and have him speak in the context of himself. The patient welcomed the healthcare team and talked about the following details of his daily life and his life history: While he often used to join in community events, he gradually quit community activities and began to communicate with people with less frequency. Regarding his daily life, he had difficulty with transportation for shopping and visiting the hospital. He needed to use a car or bus to go to the supermarket and hospital. Driving a car himself was unsafe due to his cognitive decline. However, using the bus was not convenient because the bus stop was too far from his home. Table 1 shows more comprehensive details about the patient’s social background.

**Table 1:** The patient’s social background before the action (based on the HEALTH+P assessment) Information collected after arriving at the patient's home is written in bold. † A care coordinator in the community appointed by Japanese long-term care insurance HEALTH+P: human network and relationships, employment and income, activities that make one’s life worth living, literacy and learning environment, taking adequate food, shelter, and clothing, healthcare system, and patient preference and personal values

	What: current conditions	Why: causes of current conditions	How: actions to take
Human networks and relationships	- Living alone. - Frequently calls to the fire station complaining of nonsensical things. - Bad relationship with relatives and neighborhood. - Refusal to let go of his car against the son’s advice. - Living in an apartment he owns.	- Former member of the volunteer fire department (this is why he complains to the fire station.) - Only apartments for elderly and young students standing because of urban sprawl.	-Ask the patient's son about what happens and what are matters of concern. -Recommend home care services to support the patient's life and alleviate loneliness.
Employment and income	- Former firefighter, real estate agent, and apartment landlord. - Not working now. - Adequate income from apartment rentals.	- Formerly running a real estate business on the shopping street.	-Continue monitoring the patient in collaboration with a care manager.^†^
Activities that make one’s life worth living	- "I have no pastime" saying in an examination room. - Former do-it-yourself lover. - Spending almost all his time staying at home.	- Former active participant in festivals and other town events. - Decrease in communication because of his friend's death.	- Gather information about the local communities and activities. - Actively invite the patient to events held in the local community.
Literacy and learning environment	- Declined cognitive function. - Nonadherence to the doctor's instructions. - Abundant wisdom concerning his profession and daily living. - Being not adept at learning new knowledge.	- Limited ability to get more knowledge about his medical condition. - No adviser about his self-medical care.	- Repeatedly explain his condition in an easy-to-understand manner. -Appreciate the value of the patient’s life so far.
Taking adequate food, shelter, and clothing	- Access barrier to public transport (e.g. far from bus stop). - Former hospital shuttle bus user, but recently unable to use it. - Using food delivery services once a day.	- The patient frequently experienced missing the bus because he was not able to hear the phone ring.	- Recommend the patient to use home care services. - Ask the bus staff to continue to provide support. -Introduce a w.alking assist device and home-delivery service
Healthcare systems	- Medical Care System for Older Senior Citizens. - The patient’s experienced care manager has a good relationship with the patient.	- Pride in having been self-employed and done everything himself since young, and then finding support services unnecessary.	- Recommend the patient to use long-term care services - Share the patient’s information with the care manager regularly.
Patient preference/values	- When the patient comes to the hospital, the medical staff cannot listen to his preferences or values because the patient usually tells only what he would like to tell, which is usually his worry about missing the return bus. - “I don’t like driving a car, but I must drive because I cannot go to shopping on foot partly due to the leg edema.” - “I have a strong desire to live independently, but I feel anxious about my life in the future.”		

Moreover, even the hospital courtesy bus system, which called the patient to arrange for pick-up when the bus was close to a boarding station near his house, was not convenient for him because he could not notice the phone ringing due to his hearing loss. Therefore, he was reluctant to go out of his home and frequently missed his regular follow-ups.

The interdisciplinary team had discussed how to approach this patient on a regular basis, considering his social conditions. Eventually the healthcare team had come up with the following five actions to alleviate his social problems and assist his daily life: (i) staying apprised of his thoughts, needs, and concerns by keeping in touch with him; (ii) supporting his participation in community events as he used to; (iii) introducing to him a walking assist device and home delivery service for easy shopping and other daily errands; (iv) making plans for him to continue routine outpatient visits without interruption, for example, asking the hospital bus staff to help the patient not to miss the bus (Table 1).

These actions, through transdisciplinary collaboration, changed the patient’s social conditions. He began to feel comfortable at the hospital because he could easily find someone to talk to. He came to visit the hospital regularly because the bus staff picked him up by bus or car when the patient missed the regular bus. He also became able to participate in community events held by a collaboration between residents and the hospital. In his daily life, shopping became more accessible for him because of a delivery service and an electric wheeled walker (Table 2).

**Table 2:** The patient’s social background after the action

	Patient’s condition
Human network and relationships	- Increasing trust in his care manager and the healthcare staff. - No complaint to the fire department. - Increasing communication with local residents. - Introduction of more home care services.
Employment and income	- Continuing observation of the patient’s economic condition.
Activities that make one’s life worth living	- Regular participation in a community event.
Literacy and learning environment	- Improved treatment adherence because of strengthened patient-professional relationships.
Taking adequate food, shelter, and clothing	- Shopping by four-wheel drive assisted walker. - Purchased goods delivery service from the supermarket.
Healthcare systems	- Introduction of visiting pharmacists to the patient to improve adherence. - Decreased bus missing because the hospital developed a system for him to be able to continue his hospital visits (e.g., calling the day before the hospital visit, contacting the patient to arrange for a pickup if he missed the bus.) - Reinforcement of team-based medical follow-up. For example, the patient was immediately admitted and received treatment when he developed an exacerbation of chronic heart failure.
Patient preference/values	- “I can let go of his car because my anxiety toward shopping is resolved.” - “I WILL live at home as much as possible.”

These changes resulted in the substantial improvement of his refractory edema without the use of additional drugs. Although he still lived alone, his increased involvement in the community and acceptance of care services stabilized his physical and social conditions.

## Discussion

In this case, the health and life condition of the patient changed for the better based on the assessment of his social background and the actions taken according to the assessment. In delivering personalized interventions for older patients, healthcare professionals must attend to SDH [10]. Given the often high proportion of patients with unmet social needs, screening for SDH is crucial and may impact the quality of geriatric care [11]. A previous study suggests that screening for SDH may foster healthcare professionals' respect and empathy toward their patients and then enhance the quality of care [12]. Through this case, we have learned the four important values of asking about and assessing patients’ social conditions.

First, tool-guided assessment was helpful in identifying social difficulties and unapparent challenges for the patient. Comprehensive approaches can facilitate earlier detection of patients’ social problems. However, screening SDH against patients’ wills can sometimes have unintentional negative effects on patients [13]. Therefore, healthcare professionals must keep in mind the "patient preference and values" in the HEALTH+P framework.

Second, discussion on patients’ social conditions among healthcare professionals facilitated the sharing of experts’ comments and necessary actions. Interprofessional collaboration is essential to addressing SDH in daily clinical practice [14]. This case might help improve the potential of our healthcare team.

Third, repeated assessment was vital for adaptive actions to patients’ changing biological, psychological, and social conditions. In this case, the patient did not say much about his social background until our healthcare team visited his home. The quality and quantity of communication with patients depends on what relationship patients and medical professionals have and in what setting they encounter. We learned from this case that one-time screening of patients’ social conditions may be insufficient for understanding their backgrounds and thoughts.

Fourth, negative emotions in healthcare professionals toward the difficult patient encounter could be ameliorated by a deeper understanding of the patient’s social background. Patients with unmet social needs are often mistakenly seen as irritable and arrogant people who lack sufficient communication skills, and family physicians can feel stressed by seeing such patients [15]. This negative influence may be weakened by healthcare professionals having a high capacity to address the social needs of their patients [16]. In this case, the assessment of patients’ social conditions stimulated collaboration, appropriate use of power, and empathy in the team [17].

## Conclusions

SDH assessment helps healthcare professionals become aware of patients’ social difficulties and perform efficient and effective approaches toward understanding patients’ true challenges. Recurrent SDH assessment and interprofessional collaboration should be underscored to perform comprehensive care in the primary care setting. SDH assessment may also ameliorate negative emotions toward patients with social difficulties and strengthen relationships between patients and healthcare professionals.
